# Expanding access to rehabilitation using mobile health to address musculoskeletal pain and disability

**DOI:** 10.3389/fresc.2022.982175

**Published:** 2023-01-06

**Authors:** Mathew J. Shayo, Pendo Shayo, Kelvin F. Haukila, Katherine Norman, Colleen Burke, Kennedy Ngowi, Adam P. Goode, Kelli D. Allen, Vivian Timothy Wonanji, Blandina T. Mmbaga, Janet Prvu Bettger

**Affiliations:** ^1^ Kilimanjaro Christian Medical Center, Kilimanjaro Christian Medical University College, Moshi, Tanzania; ^2^ Department of Population Health Sciences, Duke University School of Medicine, Durham, United States; ^3^ Department of Veterans Affairs Health Services Research & Development Service, Durham, NC, United States; ^4^ Kilimanjaro Clinical Research Institute, Moshi, Tanzania; ^5^ Department of Orthopaedic Surgery, Duke University School of Medicine, Durham, United States; ^6^ Thurston Arthritis Research Center, University of North Carolina Chapel Hill, NC, United States; ^7^ Ministry of Health, Community Development, Gender, Elderly and Children, Dodoma, Tanzania; ^8^ Department of Health and Rehabilitation Sciences, Temple University College of Public Health, Philadelphia, United States

**Keywords:** rehabilitation, access, mobile health, musculoskeletal disease, disability

## Abstract

**Introduction:**

Musculoskeletal (MSK) disorders such as low back pain and osteoarthritis are a leading cause of disability and the leading contributor to the need for rehabilitation services globally. This need has surpassed the availability of trained clinicians; even in urban areas where services and providers are thought to be more abundant, access can be challenged by transportation options and financial costs associated with travel, care and lost time from work. However, continuing standard of fully in-person rehabilitation care for MSK-associated pain and disability may no longer be necessary. With increased ownership or access to even a basic mobile phone device, and evidence for remote management by trained clinicians, some individuals with MSK disorders may be able to continue their rehabilitation regimen predominantly from home after initial evaluation in primary care or an outpatient clinic.

**Methods:**

This manuscript describes application of a framework we used to culturally and contextually adapt an evidence-based approach for leveraging digital health technology using a mobile phone (mHealth) to expand access to rehabilitation services for MSK-associated pain and disability. We then conducted a multi-level analysis of policies related to the adapted approach for rehabilitation service delivery to identify opportunities to support sustainability.

**Results:**

Our study was conducted in Tanzania, a lower-middle income country with their first National Rehabilitation Strategic Plan released in 2021. Lessons learned can be applied even to countries with greater infrastructure or fewer barriers. The seven-step adaptation framework used can be applied in other regions to improve the likelihood of local mHealth adoption and implementation. Our practice and policy assessment for Tanzania can be applied in other regions and used collaboratively with government officials in support of building or implementing a national rehabilitation strategic plan.

**Conclusion:**

The work described, lessons learned and components of the plan are generalizable globally and can improve access to rehabilitation services using mHealth to address the significant and increasing burden of disability.

## Introduction

Musculoskeletal (MSK) disorders—conditions that affect muscles, bones, joints and periarticular structures—are a leading cause of years lived with disability (YLDs) (1, 2). These disorders experienced by 1.71 billion people globally in 2019 are commonly classified into broad categories including low back pain (LBP) which contributes to the highest burden of MSK (36.8%), followed by other MSK disorders (21.5%), osteoarthritis (OA, 19.3%), neck pain (18.4%), gout (2.6%), and rheumatoid arthritis (RA, 1.3%) (3, 4). MSK disorders are characterized by increased pain and stiffness, with reduced mobility, dexterity, and level of functioning. This in turn can restrict self-care, home care, work and leisure activities, resulting in loss of independence, poor physical- and mental-health and reduced quality of life (5, 6).^.^ Exercise is considered the cornerstone in management of MSK-associated problems and is sometimes best supported with other physical modalities, psychosocial treatment, and multidisciplinary rehabilitation (7). Exercise programs in clinic and at home are very effective for MSK rehabilitation; however, strategies are desperately needed to address barriers to care (7, 8).

MSK disorders are the highest contributor to rehabilitation needs for every age group (3). Unfortunately, there are significant gaps between the need for rehabilitation and its availability, access, and use (9). Many low- and middle-income countries (LMICs), as well as some lower resourced and rural areas of high-income countries have limited availability of rehabilitation human resources (10–12). This occurs for many resources including issues stemming from the number of training programs, employment options and retention. Available services in urban areas and larger hospitals can introduce additional transport and financial challenges for patients to access appropriate services particularly from more rural regions (13). Even in areas with adequate service availability, healthcare providers and policy-makers are largely unaware of the evidence supporting specialized exercise programs and referrals are not made to rehabilitation providers who can address pain severity and improve physical function. The World Health Organization (WHO) recognized these challenges and with their Rehabilitation 2,030 initiative called upon stakeholders including government leaders and policy makers to assess, plan, monitor and implement strategies that expand and strengthen rehabilitation services to all who need them (14).

The United Republic of Tanzania was one of many countries that committed to Rehabilitation 2,030 and in 2021 released their National Rehabilitation Strategic Plan (15). Tanzania is identified by the World Bank as a lower-middle income country (new status as of July 2020), with a population of 58 million people who on average complete 6.3 years of education and in 2017 had a life expectancy of 68.9 years for females and 64.6 years for males (16, 17). Global burden of disease data suggest that there is a significant unmet need for rehabilitation in Tanzania, and current health trends project growing needs for rehabilitation health care (15). Priority areas identified by the Ministry of Health for strengthening rehabilitation in Tanzania are a reflection of the WHO health system building blocks (e.g., finance, workforce, service delivery) and several areas of action were immediately possible. In this practice and policy paper we use examples of efforts in Tanzania to report on a multi-level analysis of the scalability of digital health to expand access to rehabilitation. Our primary purpose was to present the translation and adaptation of an evidence-based care model that leverages mobile health (mHealth) to expand access in geographic areas with limited rehabilitation professionals for the management of chronic MSK-associated pain and disability. We then discuss the policies that should be considered for widespread adoption and sustainability. Policies were identified that could improve access to rehabilitation from the perspective of the individual with a MSK disorder. This paper is intended to serve as a foundational framework to support other regions and countries with their analysis for implementation and scale-up of mHealth in other medically underserved areas with the intent of reducing the burden of chronic MSK pain and disability.

## Evidence base and adaptation of current practice

### Current context

In Tanzania, we observed that individuals with MSK pain or reduced ability to complete daily activities initially see a general physician for medical guidance or pharmacist/medication dispensary to obtain pain relief medication. Direct access to physiotherapy is appropriate and possible but less common, due to a lack of awareness on the part of clinicians and clients that this practice is available (15). In 2021, 500 physiotherapists were estimated to be practicing in the country, which translates to less than one therapist (0.9) per 100,000 Tanzanians (18). For comparison, there are 168 physiotherapists per 100,000 people in Sweden and 105 physiotherapists per 100,000 persons in Brazil. Access to rehabilitation care was periodically restricted during COVID-19 and no form of digital health (e.g., telehealth or mHealth) was approved or implemented (19). Mobile phone use, however, continues to increase in Tanzania and serves as a potential strategy for expanding access to care. In 2020 there were over 48 million Tanzanian mobile phone subscribers with active use for daily services such as banking (20). This creates the opportunity in rehabilitation for leveraging mHealth, defined as the use of mobile and wireless technologies for health, and involves different communication channels including one- or two-way short message service (SMS), applications (apps), and mobile phone calls targeted to healthcare clients/patients or professionals (21). In alignment with the Ministry of Health's priority for evidence generation, we expanded existing partnerships to newly include a US-Tanzania focus on rehabilitation and secured funding to study implementation.

### Care delivery and adaptations

We used principles from implementation science to examine rehabilitation as a health care service in Tanzania, the gaps, and the needs and capacity for mHealth to manage MSK pain and activity limitations remotely to people's home or local community. Through a series of seven steps over 20 months from 2020 to 2022, we tailored evidence-based interventions to meet those needs. Although many frameworks exist for the adaptation of evidence-based interventions (22), we integrated components of two different frameworks: those developed by William et al. (23) and Card et al. (24). The Step Framework by Card et al. (24) was selected for its detailed processes for review and adaptation of not only the program model but also the materials for use in a new context. The Cultural Adaptation Framework by Williams et al. (23) was the only framework in the scoping review that was used effectively in international settings and provided guidance to explicitly examine differences in culture from where the interventions had been developed and tested to where they would be implemented. Integrating these intervention adaptation frameworks helped us more fully examine the culture, appropriateness, applicability and feasibility of introducing mHealth into rehabilitation care in a LMIC based primarily from evidence established in high income countries.

We began with Williams et al.'s model (23) for cultural adaptation of interventions focused on assessing community needs and context, in addition to engaging with stakeholders and experts throughout the process. This foundation (Step 1: Engaging Stakeholders and Step 2: Assessing Population and Context) were revisited with each remaining step in the process in order to build trust in the collaboration, understanding across partners and cultures, and awareness of the changing landscape of health service delivery from societal influences (e.g., politics, pandemic, policy reform). The remaining steps in our process follow Card et al.'s step framework (24) for adapting evidence-based interventions (Table 1).

**Table 1:** Process for cultural^w^ and contextual^c^ adaptation of evidence-based MSK rehabilitation care interventions.

Steps	Framework component	Examples of associated activities
1^w^	Engage stakeholders	Regular planning meetings among implementation team consisting of clinical experts from Tanzania and the United States, content experts from original research designs, and implementation science experts. Consultation with Tanzanian Ministry of Health, hospital administrators and director of Kilimanjaro Clinical Research Institute. Selected Tanzanian based champions consisting of department head, clinicians, and local researchers
2^w^	Assess population & context	Grey- and peer-reviewed literature review to determine MSK rehabilitation needs and service models. Evaluation of specialty clinic volume for MSK and estimate from this underestimate the unmet/non-referred need. Assess service capacity and patient reach.
3^c^	Select evidence-based program	Identify interventions or care models to meet needs (geographic, financial security, literacy, etc.) We selected effective telephone coaching model and integrated evidence-based strategies for text messaging that could be used to address low back, hip and knee pain.
4^c^	Gather program materials	Diagram current care model and effective intervention for adaptation. Obtain forms and formats for documentation, measures used, exercises, patient education materials.
5^c^	Identify core component	Review goals and processes of programs. Extract details from studies of text message follow up care. Discuss all program components and supporting evidence for them.
6^c^	Compare program and new context	Integrate stakeholders for cultural adaptation of process and determine the resources necessary to alter mHealth intervention components for delivery in Tanzania population. Align specific model components (e.g., text messaging) with what the elements of current practice it would address (e.g., reminders, self-reported outcome of adherence). Assess access issues for all model components and how they will be addressed. Confirm appropriate outcome measures that could indicate effectiveness of care and that can be assessed *via* mHealth (see Figure 1).
7^c^	Adapt for new context	Train staff. Make adaptations. Create and finalize patient facing materials. Test model and handoffs. Establish data monitoring and improvement plan. Confirm escalation protocols (patients who need a higher level provider for next interaction).

Abbreviations: C, Card's framework; W, William's framework.

### Evidence-based intervention

Step 3, Selecting an Evidence-based Program, focuses on selecting an effective and suitable intervention. Suitable had to also consider most accessible to the general population. Coaching by telephone is the most common application of mHealth for MSK rehabilitation. We selected a home-based exercise program that included exercise counseling by phone that was tested with older adult Veterans with LBP in the United States (25). The approach to rehabilitation was team-delivered by a physiotherapist and health coach to combine the clinical and behavior change expertise, respectively. It included a core set of strengthening and stretching exercises, and regular aerobic activity to cover major muscle groups and functional tasks. Monthly calls from the physiotherapist were used to assess potential issues and make necessary modifications in the individual's exercise program. Weekly calls from the health coach supported adherence, motivation and problem-solving barriers. Telephone-based support was well received in the United States and patients completed an average of 77% of calls. The home-based program in the United States was safe and effective for improving physical function [based on outcomes from a Timed Up & Go (TUG) Test and PROMIS Health Assessment Questionnaire].

This home-based model that was developed and tested in the United States was attractive for application in Tanzania as it did not require the rehabilitation patient and provider to meet frequently in person (initial evaluation and study follow-up without intervention at 90-days) and it extended the rehabilitation workforce by including someone trained in exercise and behavior change (a Tanzanian rehabilitation strategic plan priority). It was determined that the primary outcomes (TUG and PROMIS) and secondary outcomes (Satisfaction with Physical Function, the Patient-Specific Functional Scale, and the Roland-Morris Disability Questionnaire) from the research in the United States (25) would be important and feasible to measure in the Tanzania-based model. After a review of the intervention protocol tested in the United States, and thorough evaluation of the standards of care in Tanzania, including workforce, service delivery, and information documented, we moved to Step 4, Gather Program Materials, from both countries.

### Text messaging

Telephone coaching as an alternative to in-person care can improve access to care for some people and for others can still be expensive and geographically out of range. Pay per use mobile phones can generate large expenses with per minute rates and roaming charges. This necessitates the evaluation of additional options. The next most accessible and low-cost approach was to consider text messaging. The feasibility, acceptability and effectiveness of SMS texts on simple (e.g., flip) mobile phones in LMICs has been demonstrated for several populations and use cases. Examples include improved childhood immunization coverage (26), antiretroviral therapy adherence (27–29), and clinic appointment attendance (30). Studies also demonstrated a reduction in personnel resources and the opportunity to assess adherence. In order for us to propose to incorporate SMS texts to support MSK rehabilitation adherence, we moved to Step 5, Identify Core Components, specific to the text messaging.

Text messages supporting exercise, physical activity or rehabilitation interventions for adults with MSK conditions (31–38) (Table 2) included cues for behavioral change (37, 38), exercise/physical activity assessments of adherence (31, 32, 34, 35), and reminders (36). One study sent texts daily (34), five studies sent weekly texts (31, 32, 35, 37, 38), and some at participants' indicated preferred time (34). Based on available evidence, resources and expertise, our model for Tanzania will include text reminders to complete prescribed exercises, patient-reported completion (to measure adherence) and positive reinforcement for affirmation. Considering the state of the science, we will incorporate patient feedback when implemented to modify text timing and frequency. Decisions in other regions or countries may be further influenced by whether mHealth for rehabilitation is planned for integration in primary care vs. outpatient specialty clinics.

**Table 2:** MSK exercise, physical activity or rehabilitation interventions that informed Our design for incorporating text messages .

Author, year	Interventionist	Intervention design (comparison)	Frequency of text messaging	Characteristics of text messaging	Content of text message (is it informed by behavioral training)	Example
Bennell, 2020 (31)	Not reported	Text message vs. no text message	– Text messages were sent each week (weeks 1–8) to fortnight (weeks 9–24). – Average number of messages sent to participants is 46–58 for 24 weeks	Automated, semi-interactive message. One-way, two-way message	– Prompt for self-report of exercise sessions in the past week – Positive reinforcement – Barrier identification – Barrier Behavior change technique (BCT) – Facilitator BCT – Reminder to respond – Response not supported – Opt out – Special occasion	Example: “Hi (name) How many days did you manage to do your home exercises this week? Please reply with ONLY ONE number between 0 and 7”
Blake, 2015 (32)	Health psychologist and a medic	Arthritis Research UK information booklet (about knee OA and exercise) + physical activity diary + Text messages	4 PA promoting messages/week over a period of six weeks. Frequency based on evidence for one message/week required to promote behavior change and preferences expressed by the group.	Automated system	– Importance of physical activity for general health and knee OA – Practical suggestions for ways of increasing physical activity alongside knee OA	Example: “Interesting fact: as well as helping to manage your arthritis, being physically active helps reduce the risk of osteoporosis, diabetes and some types of cancer!”
Campbell, 2019 (33)	Not reported	Usual care (perioperative education) + Text messages and video message vs. Usual care	93 text messages over a 6-weeks delivered based on patients’ recovery progress	Automatically sent through the surgeon's SMS bot (StreaMD) was hosted on a health insurance Server.	– Recovery instructions – Encouraging and empathetic statements.	Example: Motivation—“Pain is normal after replacement surgery. It can be specifically intense during the first few days after…”
Chen, 2017 (34)	Research assistant trained by physician	Exercise + exercise materials (printed pamphlet) + Text messages vs. Exercise + exercise materials (printed pamphlet)	– Daily for 2 weeks – 14 text messages – entered into text message platform in advance and sent automatically at 20:00 h or at participants’ preferred time.	Automated	– Reminders – Encouragement – Educational messages.	Example: “found at the publisher's website”
Lambert, 2017 (35)	Trial physiotherapist	App (HEP) + Phone call + Text message vs. HEP (paper handouts)	Text messages were sent weekly	Not reported	– Motivational statements	Example: “keep up the hard work” or “well done completing 4 weeks of home exercises.”
Lilje, 2017 (36)	Not reported	Exercise + Text messages	First reminder 3 days after the visit. Following reminders sent every third day for 3 weeks. Then 1x/week for another 2 weeks	One-way text messages	– Reminder text – Exercises (stretching of the ilio-psoas muscle and breathing technique)	Example: “Hello! This is a reminder of your home exercise (s)! Kindly, xxx.”
Nelligan, 2021 (37)	Study coordinator	Website + Text messages vs. Website	Self-report exercises weekly And interaction from reported activity level Twice weekly behavior change message	Automated behavioral change text messages -Encouraging exercise adherence	– Self-report – Positive reinforcement – Barrier identification – Barrier behavior change – Suggested behavior change – Reminder to respond – Error in response – Opt out – Special occasion	(See Bennell)
Thomsen, 2016 (38)	External communications consultant	Motivational counselling sessions + Text messages VS Motivational counselling session	1 to 4 reminders per week with behavioral goals and action plans.	SMS-Track Aps, (https://www.smstrack.com/)	– Reminder statements according to the goal (behavioral).	Example, Goal—Break up prolonged sitting by standing up frequently “Hi X. Anything interesting in the newspaper today? When you finish the next article, get up and stretch your legs and maybe put another log on the fire.”

### Transforming care

**Figure 1:**
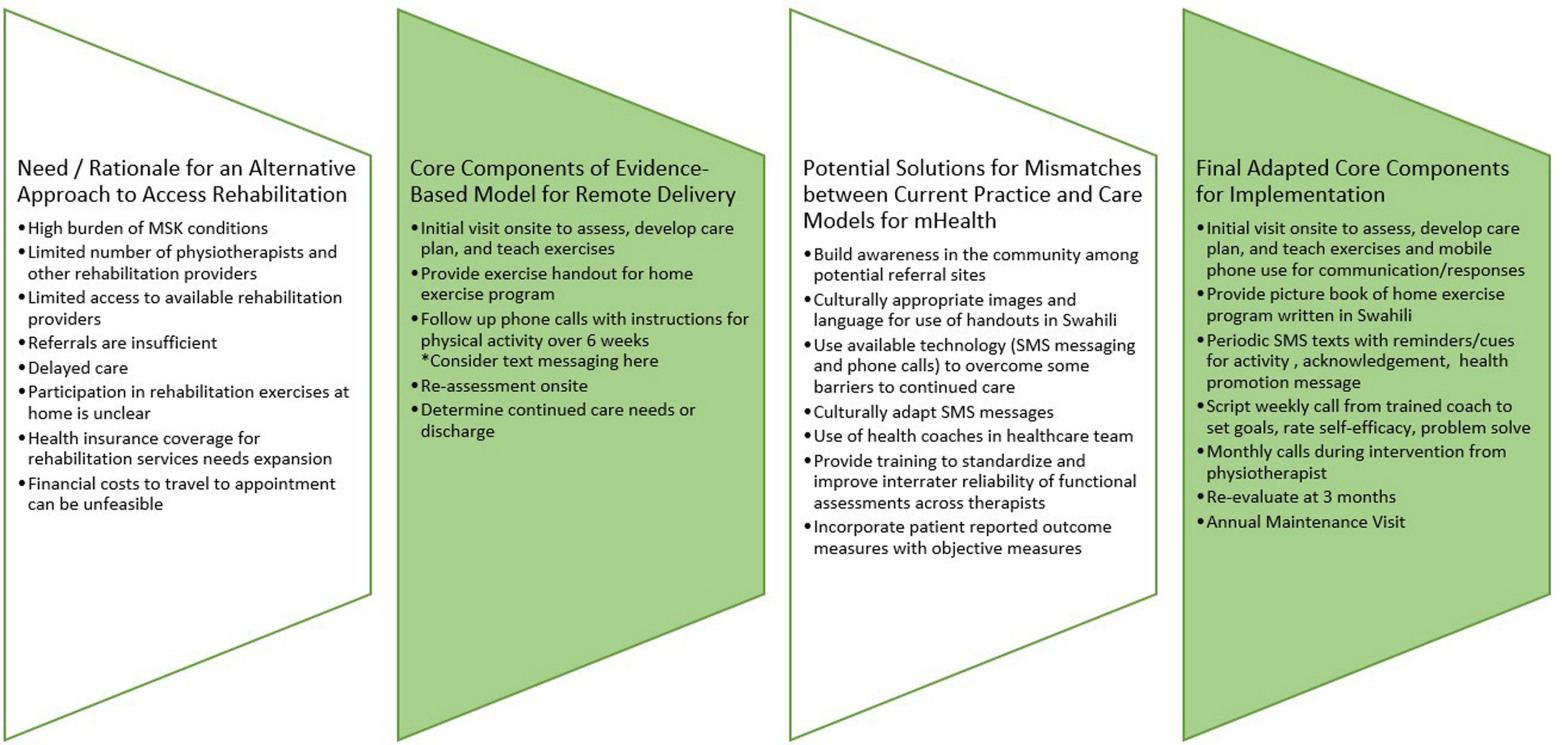
Cultural and contextual adaptation of evidence-based approach for leveraging mHealth to increase access to rehabilitation.

Our comparison of the US model to the standard of care in Tanzania (Step 6: Compare) using a framework for translation and adaptation demonstrated a rehabilitation care model that included mHealth would be feasible with some adaptations (Step 7: Adapt for New Context and Figure 1). Digital health and options for mid-level providers are two of Tanzania's Rehabilitation Strategic Plan “Areas of Action” for increasing the availability of rehabilitation services and expanding the workforce. However, neither existed for rehabilitation care and would need additional cycles of adaption. Applying the seven-step adaptation framework in iterative cycles prepared us for implementation that begins in 2022. Future sustainability may depend on policies.

## Policies for scalability

**Figure 2:**
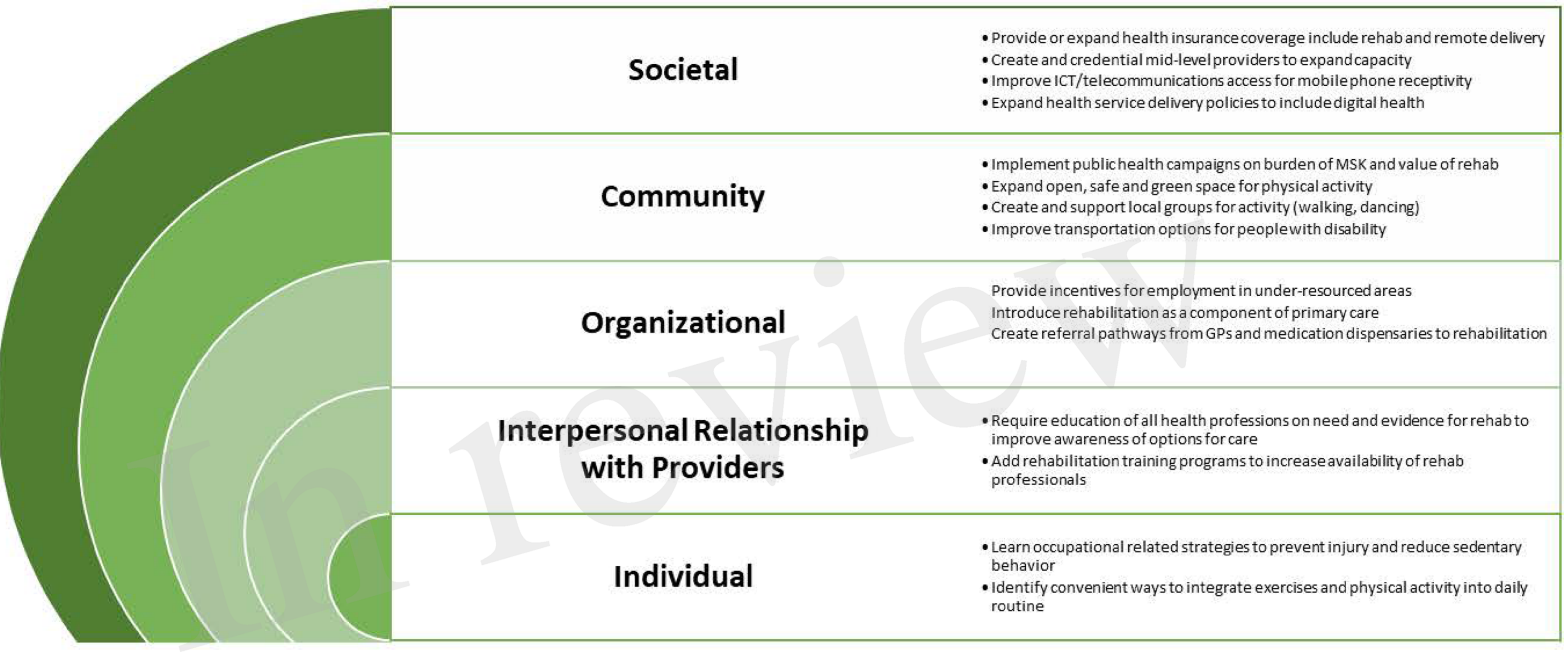
Policies at multiple levels that could influence individual access to rehabilitation that includes mHealth for MSK.

We felt an assessment of policies to support scalability of these strategies to improve access to rehabilitation was important for understanding influential factors at multiple levels. Some but not all countries have an active national rehabilitation strategic plan where government leaders have proposed strategies with policy reform. For this analysis we evaluated the policy landscape for areas that would be considered priorities from the perspective of the individual rehabilitation recipient (patient or client). Using ecological systems theory that views an individual's behavior as a component of multi-level relationships in a system, we identified policies at the interpersonal, organizational, community and public policy levels (Figure 2) (39). The individual levels are as important as the reciprocal relationships between them. For example, working directly with the patient (individual level) and establishing policies that shape the skills and attitudes of providers (interpersonal level) are each important; taken together, a health care provider's understanding of the evidence for rehabilitation in addressing MSK-associated disability will have an effect on patient's next course of action. Related, as more healthcare providers understand the need for rehabilitation services (interpersonal), it is possible that more referral pathways will emerge from many more healthcare disciplines (organizational level) which could lead more for patients to be considered for rehabilitation. Reciprocity between the person and the environment, and between levels of the environment is dynamic and will shape and influence different levels over time.

In Tanzania, several key policies are identified in the rehabilitation strategic plan that patients may not consider directly related to their care but are vitally important for building capacity and strengthening rehabilitation services as part of the healthcare and public health infrastructure. Obtaining a rehabilitation-specific budget line item in the Ministry's administrative structure is needed to support resourcing and sustaining any action of the national rehabilitation strategic plan. Related, integrating rehabilitation data in to the Health Management Information System is needed in order to establish national indicators and an evaluation plan for improvement. These same areas of budgeting and documenting need to be addressed at local levels. Many times local needs will need local solutions and data and finances to support change will be paramount.

## Conclusion

We propose that in conjunction with national rehabilitation strategic plan development and implementation, that an evaluation of local practice with best available evidence and consideration of policies at multiple levels could stimulate changes to improving access to rehabilitation. In our work, we found that adapting evidence-based approaches to support rehabilitation at home using telephone calls and SMS text messaging *via* mobile phones could expand the capacity for services and number of people with rehabilitation needs treated. International partnerships helped facilitate this work and could serve as a catalyst or accelerator in other regions. Timelines and priorities can change over time and communication between activities locally and nationally could help spread successful strategies. Data clearly illustrate that rehabilitation needs will only continue to escalate with the trends in population aging and activity limitations from long-COVID. Leveraging mHealth to expand access to rehabilitation could be an essential strategy for reducing the burden of disability.

## Data Availability

The original contributions presented in the study are included in the article/Supplementary Material, further inquiries can be directed to the corresponding author/s.
